# Differences in Motivation to Engage in Sexual Activity Between People in Monogamous and Non-monogamous Committed Relationships

**DOI:** 10.3389/fpsyg.2021.753460

**Published:** 2021-11-03

**Authors:** Anna (Kelberg) Kelberga, Baiba Martinsone

**Affiliations:** Department of Psychology, University of Latvia, Riga, Latvia

**Keywords:** monogamy, non-monogamy, sexual activity, reasons to engage in sex, reasons for sex

## Abstract

This study compared motivations to engage in sex between monogamous and non-monogamous respondents (*n* = 1,238, out of which 641 monogamous and 596 non-monogamous respondents; women—47.4%, men—50.9%, other gender—1.7%; age: *M* = 27.78 years, *SD* = 7.53, range = 18–62). The research aim was to identify whether there are differences in self-reported reasons to engage in sexual activity between these two groups. Presented with 17 reasons to engage in sexual activity, the respondents rated the frequency with which they engage in sex for each reason. While both monogamous and non-monogamous respondents reported to engage in sex with the same frequency for the reasons of sexual release and physical desirability of a partner, the research uncovered that non-monogamous respondents engage in sex significantly more often to seek new experiences, to boost self-esteem, to guard their mate, to have a specific kind of sex (such as anal, kink, or fetish) or to experience the thrill of the forbidden. Non-monogamous respondents reported higher frequency to engage in sex for most reasons overall. The study also revealed that there are differences in reasons to engage in sex among men and women, some of the differences are related to the relationship status (monogamous or non-monogamous), while others are universal for men or women irrespective of their monogamy status.

## Introduction

People’s sexual arrangements are complex and neither monogamy nor non-monogamy can be considered the norm. Instead, both serve certain needs to maximize social, economic, and relational benefits (Wood et al., 2018). The aim of this research is to gain a deeper understanding of reasons that motivate people to engage in monogamous or non-monogamous sex.

Although it is tempting to classify romantic relationships into strict frames of monogamy and non-monogamy, reality is nuanced and complex. For instance, an initially monogamous relationship may become non-monogamous when one of the partners cheats, or possibly transform into consensually non-monogamous relationship if partners agree, or become asexual due to the loss of trust (Nelson, 2013). This is to say that boundaries between different forms of relationship structures are blurry (Richards and Barker, 2013) and should be perceived as a generalized trend that may have various exceptions and overtones.

### Monogamy

A relationship in which the explicit agreement or taken-for-granted rule is that there should not be any sexual or romantic relationships outside main partnership is called monogamous (Richards and Barker, 2013). Fisher (2011) states that pair bonding is universal for humans and most men and women are willing to get married. Even as worldwide marriage rates have declined, still 85–90% of modern Americans intend to get married (Cherlin, 2009) and European marriage rates are in a similar range, especially if “consensual unions” (a “marriage-like” relationships) are taken into account (Eurostat, 2015). Fisher (2011) adds that although polygyny, a practice of mating with multiple wives (Gould et al., 2008), is permitted in 72–84% of human societies, only 5–10% of men in these cultures actually have several wives simultaneously, which leads her to conclude that across the cultures the dominant pattern is to marry one person at a time.

Although the concept of monogamy seems straightforward from the first sight, there is high degree of variation. The umbrella term of monogamy includes both *lifelong monogamous* partnerships, meaning one sexual partner across the lifespan (Pinkerton and Abramson, 1993) and *serial monogamy*, meaning several mutually monogamous, non-concurrent partners across the lifespan (Pinkerton and Abramson, 1993; Fisher, 2011). Although lifelong monogamy may be particularly beneficial from a risk-reduction perspective (e.g., it would decrease the chances of sexually transmitted infections), this form of monogamy is rare and represents about a quarter of population (Conley et al., 2012b). One study has found that men have six to eight sexual partners and women report about four sexual partners by the age of forty—a indication that most people do not adhere to monogamy in its strictest sense (Mosher et al., 2005). Divorce and remarriage are not uncommon and are presented across cultures (Fisher, 2011). This is well illustrated by a study that found that 10% of women have had three or more husbands by age 35 (Cherlin, 2009). In the modern Western societies, monogamous relationships are predominant and mostly desired form of intimate partnership arrangements (Dindia and Emmers-Sommer, 2006; Finkel et al., 2014; Impett et al., 2014). At the same time divorces are not uncommon—for many decades, about half of Western marriages end in divorce (Brandon, 2011). Of the marriages that remain intact, it has been estimated that about 20% are sexless (Weiner Davis, 2003).

In line with common practice this study will use the definition of monogamy primarily addressing sexual monogamy in the current relationship (either lifelong or serial), assuming that when most people refer to monogamy, they are referencing sexual commitments (Conley et al., 2012a) vs. “social monogamy,” which is sometimes called “marital monogamy” and refers to marriage of only two people at a time (Reichard and Boesch, 2003).

A study on expectations regarding partner fidelity by Watkins and Boon (2016) found that people in relationships are not indifferent to infidelity (the mean rating on the importance of fidelity item was *M* = 6.75, *SD* = 0.73 on a 7-point scale) and the vast majority indicated that they would want to know if their partners had cheated on them. A study on couples who are married less than 10 years suggests that people in early stages of their marriage estimate probability that their spouses would ever engage in extra-marital sex as 7, 9% and more than a third reported that there is no chance that their spouse would cheat on them (Wiederman and Allgeier, 1996). Another study by Buss and Shackelford (1997) asked the same question to newlyweds, who estimated that there is less than 3% chance that their spouses would engage in one-night stand, a brief affair or serious affair in the next year. Individuals in committed dating relationships estimated probability of infidelity by their partner in the similar range, roughly 5–9% (Watkins and Boon, 2016). However, when people are asked about likelihood of infidelity among other people, not regarding their own partner, they estimate that there is about 42% chance that an average person of the opposite sex is cheating on his or her partner (Watkins and Boon, 2016). This finding shows that although people significantly underestimate their chances to be cheated on, they see infidelity as reasonably common and general estimate is consistent with statistics on reported infidelity rates.

Nelson (2013) says that many couples may assume that they are monogamous (“implicit monogamy”), but never discuss exactly what the monogamy agreement means to them (“explicit monogamy”). A research by Warren et al. (2011) indicates that only 52% of couples have explicit agreement on monogamy and 71% of those sustain the agreement, 40% of couples find that their expectations about what monogamy means to each of them are significantly different. Nelson (2013) invites to look at the monogamy from various perspectives, rather than a dichotomous term, and defines various facets of monogamy (including, but not limited to: thoughts, fantasy, sex, love, flirtation, etc.) and advises couples to define their monogamy expectations in each facet.

To sum up, monogamy is the prevalent mating strategy for humans. Though many relationships are monogamous rather in name than in deed (Duncombe et al., 2004)—monogamy in its classical manifestation is rare and many relationships includes a degree of non-monogamy.

### Non-monogamy

Non-monogamous arrangements practiced by humans are elaborate and nuanced. Mogilski et al. (2017) states that non-monogamy exists in a variety of forms across cultures, including serial monogamy (there is no unified view whether serial monogamy should be seen as a form of non-monogamy or not), polygyny (i.e., the marriage of one man to two or more women), polyandry (i.e., the marriage of one woman to two or more men), polygynandry (i.e., group marriage), non-consensual non-monogamy (i.e., infidelity), and consensual non-monogamy (Loue, 2006).

Some authors suggest that viewing monogamy and non-monogamy as binary opposites might be misleading and simple dichotomy might be insufficient to explain relationship differences (Parsons et al., 2013; Ferrer, 2018) and recommend looking at it as a continuum along which relationships can be defined. Some authors distinguish those who identify as monogamous, monogamish (a relationship defined by some degree of openness to sexual/emotional relationship outside the couple; Berry and Barker, 2014), and consensually non-monogamous (Parsons et al., 2013), while others propose so called “nougamy” as a rejection of mono/poly binary (Ferrer, 2018). This study does not investigate polygyny, polyandry, and polygynandry as these forms of non-monogamy are very uncommon and mostly illegal in the Western word (Tucker, 2014).

### Non-consensual Non-monogamy

Researchers suggest that humans have evolved a dual reproductive strategy—along with almost universal tendency to form pair bonds (Guitar et al., 2017) there is a universal tendency to engage in adultery (Fisher, 2011). Brandon (2016) adds that individual sexual strategy is influenced by a combination of person’s personal, relationship, environmental, and cultural background. Non-consensual non-monogamy is any type of sexual behavior outside the current relationship that violates the explicit or implicit sexual monogamy norms and is associated with feelings of betrayal (Buunk and Dijkstra, 2004; Barta and Kiene, 2005). These include, but are not limited to behaviors such as intercourse, oral or anal sex, or online sexual activities (Braithwaite et al., 2010).

Some authors further distinguish among sexual and emotional infidelity (Buss et al., 1992; Guitar et al., 2017; Rodrigues et al., 2017). Guitar et al. (2017) states that across cultures women and men have universal understanding of sexual infidelity. In one study where respondents were asked to define what is sexual infidelity, most responded in the same way: “Sexual activity with an individual other than one’s partner.” At the same time conceptualization of emotional infidelity remains under investigated and influenced by other factors, including individual’s sex, culture-specific gender roles, and unique dyadic characteristics of a couple (Guitar et al., 2017). Thus, considering the ambiguous and unclear nature of emotional infidelity, for the purposes of this study a definition of infidelity by Rubel and Bogaert (2015) will be used—“having secret sex with another partner/s” and thus focusing on sexual aspect of adultery.

While engaging in sexual intercourse with someone outside of the relationship and without the primary partner’s consent is clearly cheating (if this has been established as breaking the rules of the relationship, especially in a monogamous relationship), other, more ambiguous behaviors, are more challenging to classify as cheating. Some research has focused on less-traditional understandings of infidelity. Whitty (2003) investigated perceptions of offline and online behaviors such as cybersex, hot chat, pornography use, and emotional and sexual intimacy. Porn use was seen as a factor separate from sexual or emotional infidelity and was seen as less worrisome (Whitty, 2003). In contrast, online relationships are considered by many to be as hurtful and detrimental to real-life relationships as offline infidelity (Whitty, 2003). In fact, almost equally high percentage of people considered having an Internet relationship to be an act of betrayal as those who viewed “seeing someone” offline as an act of betrayal (84 and 90% of respondents, respectively, viewed these acts as betrayal) (Schnarre and Adam, 2017). Sexual fantasies about someone other than a partner can be perceived as infidelity and provoke jealousy (Feldman Shirley and Cauffman, 1999; Yarab et al., 1999, as mentioned in Schnarre and Adam, 2017). A recent study found that 83% of respondents view sexting outside primary relationship to be cheating (Falconer and Humphreys, 2018). Parasocial behaviors (relationships with real-life celebrities or fictional characters) are less likely to be seen as infidelity compared to offline or cyber infidelity, though still perceived as a form of betrayal and potentially harmful to real-life romantic relationships (Schnarre and Adam, 2017). As to sexual fantasies, the more a person sees them as a threat to a relationship, the more it is perceived as infidelity (Yarab and Allgeier, 1998, as mentioned in Schnarre and Adam, 2017).

Men and women differ in what they define as infidelity and in how distressing they find those behaviors (Schnarre and Adam, 2017). Overall, women define a wider range of behaviors as infidelity (Whitty, 2003; Hackathorn, 2009). Though both men and women find sexual infidelity to be equally distressing (Yarab et al., 1999).

Research suggests that sexual “infidelity” is a common cause of divorce (Amato and Previti, 2003). However, Rubel and Bogaert (2015) argue that it could be the case that it is the break of trust, not non-monogamy itself, that makes sexual infidelity disruptive as, trust is believed by theorists to be an important component of relationship quality (Fletcher et al., 2000).

Regardless the facts that majority of the population gets married at least once (Olson and DeFrain, 2003; Cherlin, 2009) and most individuals in committed relationships generally disapprove of infidelity, acts of infidelity are common (Hall and Fincham, 2009; Jackman, 2015). Estimates of relationship infidelity depend on the type of methodology used, the sample surveyed, and the definition of adultery used by the researchers, yet most studies report infidelity rates in the range from 26 to 70% for women and from 33 to 75% for men (Eaves and Robertson-Smith, 2007; Block, 2008, as mentioned Nass et al., 1981; Zimmerman, 2012, as mentioned in Brandon, 2011). In a study of those who are not married, but in committed relationships, 40% of the study participants responded that they knew that their partner had cheated on them and another 19% were unsure (Emmers-Sommer et al., 2010). Some authors suggest that the number of married couples that experience an affair over the course of marriage may be as high as 76% (Thompson, 1983, as mentioned in Conley et al., 2013). At the same time surveys show that 79% of Americans of all ages believe that it is always wrong for a married person to have a sexual relationship outside of a marriage (Zimmerman, 2012).

### Consensual Non-monogamy

A relationship in which both partners explicitly agree that one or both of them may have romantic or sexual relationships with others go under the term consensual non-monogamy, commonly abbreviated as “CNM relationships” (Conley et al., 2013; Burleigh et al., 2017), or also called “open relationships” as an umbrella term (Zimmerman, 2012), “open non-monogamy” (Richards and Barker, 2013), “intentional non-monogamy” (Noël, 2006, as mentioned in Matsick et al., 2014), “responsible non-monogamy” (Lano and Parry, 1995; Anapol, 1997; Klesse, 2006; as mentioned in Matsick et al., 2014), or “non-secret negotiated non-monogamy” (Jamieson, 2004, as mentioned in Matsick et al., 2014). Zimmerman (2012) states that consensually non-monogamous relationships are different from adultery in a way that partners explicitly agree on the sexual boundaries of the relationship and there is no deception regarding sexual activity of the involved parties. According to Cohen (2016) 75% of consensually non-monogamous couples have explicit rules in their relationship regarding their non-monogamous activity.

Some authors suggest that CNM relationships among humans is not a modern-day phenomenon but have existed throughout the human history (Ryan and Jetha, 2010; Zimmerman, 2012) and, contrary to popular opinion, theoretical work in psychology does not suggest that CNM relationships are pathological (Rubel and Bogaert, 2015). Evolutionary psychology suggests that individuals engage in multiple mating strategies because different strategies are effective in different situations (Jonason et al., 2012). As individuals engage in a variety of forms of casual relationships because these relationships serve different functions (Jonason, 2013), varying forms of consensual non-monogamy in a similar way may have a strategic function (Rubel and Bogaert, 2015). Rubel and Bogaert (2015) argue that from this perspective, CNM relationships can be viewed as part of the normal range of human sexuality rather than as a symptom of an individual’s psychological or relational problem. Jonason et al. (2012) add that behaviors that are natural will not necessarily result in individual happiness or happiness within the relationship. For example, having secret extra dyadic sex (i.e., “cheating”) can be an effective strategy from an evolutionary standpoint (Jonason et al., 2012), but may not contribute the quality of an individual’s monogamous relationship (Amato and Previti, 2003, as mentioned in Rubel and Bogaert, 2015).

The prevalent forms of consensual non-monogamy present in Western cultures are swinging, open relationships and polyamory (Richards and Barker, 2013; Rubel and Bogaert, 2015). These categories are not mutually exclusive and oftentimes definitions may be inaccurate due to subtle nuances of human sexual and romantic relationships (Matsick et al., 2014). As consensual non-monogamy is a relatively understudied phenomenon, there is no firm consensus on terminology and defining characteristics of CNM forms are debatable (Grunt-Mejer and Cambell, 2016).

Matsick et al. (2014) define swinging couples as those, who engage in sexual relationships with people other than their primary partner (see below for definition of “primary partner”) and typically engage in these relationships at a party or in another social setting. Grunt-Mejer and Cambell (2016) when defining swinging, stress the same key components—extradyadic sex, usually at parties or social situations, where both partners are present. An important element of a swinging relationship is that the primary couple views swinging as something that they do together as a couple and view swinging activity as a pastime for the couple (Matsick et al., 2014). Participants of a swinging party or convention have a common understanding that they are not monogamous (Matsick et al., 2014) and may engage in different behaviors—couples exchanging partners with another couple for sexual purposes or inviting a third person to engage in sexual activities with the couple (Buunk and van Driel, 1989, as mentioned in Walshok, 1971; Matsick et al., 2014; as mentioned in Matsick et al., 2014). Swinging partners usually separate sex and love, with only sex with others being accepted (Barker, 2011). Some authors further distinguish between open and closed swinging (Serina et al., 2013). Open swinging occurs when the couple engages in sexual relations with another couple in close physical proximity to each other and closed swinging occurs when the swinging occurs in separate rooms (Jenks, 2001). There is also soft and hard (sometimes also called “full”) swinging used to describe the amount of sexual contact made between the partners involved in the swinging—soft referring to the absence of intercourse (limiting to other sexual activities, like kissing or petting) and full sexual intercourse (Serina et al., 2013). Typically, swinging is practiced by heterosexuals and bisexuals (Barker, 2011).

Polyamorous relationships are those in which not only sexual but emotional relationships are conducted with multiple partners (Matsick et al., 2014; Grunt-Mejer and Cambell, 2016). Unlike swingers, polyamorous individuals are more likely to describe their multiple relationships as having a romantic or emotional component, rather than being strictly sexual (Sheff and Hammers, 2011, as mentioned in Matsick et al., 2014). Although polyamorous individuals typically reject sexual and emotional exclusivity, these relationships often involve explicitly negotiated agreements about what types of extra-dyadic interactions are permitted by each partner (for example, spending a night together, having unprotected sex, etc.) (Wosick-Correa, 2010). Though Barker (2011) states, that many other polyamorists emphasize the importance of individual freedom, communication and ongoing negotiation and reject relationships rules.

While polyamory and swinging are well defined, the meaning of the term “open relationship” is less clear and in some publications has sometimes been used as an umbrella term for CNM relationships. The term “open relationship “is used to describe a relationship arrangement where partners seek sexual relationships independently from one another, like swingers and polyamorists, people in open relationships and their partners consent to being non-monogamous (Matsick et al., 2014).

For the last decade research on non-monogamy is on the rise (Rubel and Bogaert, 2015). Although a growing body of research has examined relationship quality among people engaged in non-monogamy (Conley et al., 2013; Rubel and Bogaert, 2015; Mogilski et al., 2017; Moors et al., 2017), it is still unclear what makes people inclined to engage in these types of relationships in the first place. In addition, general population of those who identify themselves as non-monogamous remains understudied as most of the research in this field focused on homosexual males (Cohen, 2016) and even fewer studies have systematically compared monogamous and non-monogamous relationships using quantitative analyses (Mogilski et al., 2017).

The reasons why people engage in sex are numerous and complex and are not limited by obvious reasons for reproduction, relief of sexual tension and sexual pleasure. Since the development of the original YSEX? questionnaire (Meston and Buss, 2007) that identified 142 reasons to engage in sexual activity based on the student sample, several other studies were performed to identify how these reasons change under different circumstances (see Armstrong and Reissing, 2014, 2015 for women’s motivations to have sex in casual and committed relationships with male and female partners; Wood et al., 2014 for reasons for having sex among lesbian, bisexual, queer, and questioning women in romantic relationships; and Wyverkens et al., 2018 for a replication study in different age groups). However, the reasons to engage in sexual activities were not studied from the perspective of non-monogamy—do people in non-monogamous relationships differ from people in monogamous relationships in terms of why do they engage in sex? The goal of this research was to investigate reasons to engage in sex among monogamous and non-monogamous respondents in committed relationships. The research question was to understand if there are significant differences in frequencies choosing different reasons to engage in sexual activity among monogamous and non-monogamous respondents in committed relationships.

## Materials and Methods

### Participants

The target population was defined as individuals 18 years old or older in committed relationships or married. Simple random sampling was used to collect data. To recruit a large and diverse sample of an understudied population of individuals that engage in non-monogamous relationships more than 50 ads were published online. Participants that are married or in committed relationships were recruited from social media websites, discussion groups and forums, and websites for people with specific interests. People who were not married or in committed relationship were prompted to a “thank you” page and did not participate in the survey. Some of the ads were targeted toward specific groups online with the likelihood that individuals would be willing to discuss their non-monogamous relationships (for example, forums on dating websites for swingers, forums for polyamorous individuals, etc.), while some of the ads were published on websites that are not related to sexuality (e.g., sub-Reddit forums for confessions, forums for those who are over 50 years old, etc.).

1,702 respondents have decided to participate in the research and filled in the questionnaire. In total 464 responses were excluded from the sample—mostly those who submitted incomplete responses or who indicated that have more than one sexual partner but responded only about one of their partners. Finally, data from 1,238 respondents (women—47.4%, men—50.9%, other gender—1.7%; age: *M* = 27.78 years, SD = 7.53, range = 18–62) was analyzed. Before the beginning of the survey participants were presented with a consent form and informed about data processing procedure. Further, participants answered a set of demographic questions about their gender, age, education and ethnicity. In line with recommendations by SMART (2009), participants reported their sexual self-identification (heterosexual—71.9%, bisexual—21.4%, or homosexual—2.6%, other—4.1%), sexual attraction (only attracted to females—34.6%, mostly attracted to females—16.8%, equally attracted to females and males—8.4%, mostly attracted to males—23.3%, only attracted to males—16.6%, not sure—0.3%), and sexual behavior (men only—29.4%, women only—42.5%, both men and women—28.1%). Those survey participants, who indicated that they are in married or committed relationships (only these groups were eligible for participation) chose one of the options, that best describes their monogamy relationship arrangement (in a monogamous relationship—51.85%, in non-monogamous relationship—48.15%) and reported relationship duration (5% being in a relationship less than a year, 35.3%—1–5 years, 34.4%—6–15 years, 16.3%—16–25 years, 5.1%—26–35 years and 1.9% more than 35 years). Then, respondents in non-monogamous relationships answered an identical set of 17 questions about their reasons to engage in sex with each of the partners. Monogamous respondents answered the same set 17 of questions only about their one partner. Survey participants were given an option to write their own reasons if these were not reflected in the survey questionnaire. In the end of the survey participants had an option to leave their e-mail address if they were willing to participate in further interviews.

### Procedure

Prior to advertising the survey, pilot interviews were conducted both in person and over video call by the first author of this paper. Two pilot interview participants were males, one participant was a female and one male-female couple. While conducting pilot interviews, it became clear that respondents experience difficulties responding straight away to the question of their motivation to engage in sex. Thus, interviewees were asked a broader range of questions that led to the topic of motivation indirectly, for example, asked about their understanding of monogamy, how they decided to live a non-monogamous lifestyle, who was the initiator, what was their experiences of engaging with other non-monogamous individuals and the challenges and benefits they experienced. Interviews were not transcribed verbatim, but in note form. All names have been changed and identifying details omitted, in some cases respondents’ real names were not known from the beginning. No other personal information that could identify an individual was asked. Then, a pilot questionnaire was posted online and after initial feedback, was improved, and published again. Link to the pilot questionnaire was made invalid to avoid that older version of the questionnaire is taken. One of the concerns in the pilot questionnaire was that it didn’t specify which of the partners to select if the respondents had more than two partners. Further an improved questionnaire was posted online. SurveyMonkey, an online survey services company, hosted both the informed consent and questionnaire. Participants were also asked to complete basic demographic questions. At the end of the study respondents were invited to provide their e-mail address if they are willing to participate in more detailed interviews. Responses to the question on sexual arrangements (married or in committed relationships, open relationships, swinging, polyamory, non-consensually non-monogamous, asexual) were given in a randomized order to avoid answers options being perceived in a hierarchical order. SurveyMonkey provides an option to control Internet protocol (IP) addresses so that the survey cannot be taken more than once from the same IP address. This way chances that the same respondent takes the survey twice or more were minimized.

Ethical approval for the study was granted by Ethics Committee for Humanities and Social Sciences research involving human participants, University of Latvia.

### Measures

To measure sexual motivation, a questionnaire was developed to assess frequency to engage in sexual activity for different reasons. The questionnaire developed for the purposes of this study consists of 17 reasons for sexual activity. 14 of these questions were adopted from YSEX? questionnaire (Meston and Buss, 2007). The original YSEX? questionnaire consists of 142 items, that are further grouped into 4 large factors and 13 subfactors (Meston and Buss, 2007). It takes up to 45 min to complete the original survey (Armstrong and Reissing, 2014). A questionnaire of this length would be impractical and too time consuming for non-monogamous respondents who were asked to complete identical questionnaires about two of their partners, which together with additional questions would take close to 2 h. Long surveys have lower completion rates and higher abandonment rates (Saleh and Bista, 2017). Thus, for the purposes of this study a new set of questions was developed, each representing one of the original YSEX? questionnaire’s subfactors (stress reduction, pleasure, physical desirability, experience seeking, resources, social status, revenge, utilitarian, love, and commitment, expression, self-esteem boost, duty/pressure, mate guarding). One of the subfactors (“resources”) was represented by two questions: “I wanted to get resources from that person (such as promotion, money, etc.)” and “I wanted to conceive a child” as procreation is a strong reason for having sex and tends to stand out in the literature (Leigh, 1989; Hill and Preston, 1996), but do not represent the factor by its own. This resulted in a set of 14 questions:

Stress reduction: I wanted to release stress, anxiety, tension or to fight boredom
Pleasure: I was sexually aroused or wanted to experience physical pleasure
Physical desirability: The person was physically attractive
Experience seeking: I wanted new sexual experience or to act out a fantasy
Resources: I wanted to get resources from that person (such as promotion, money, etc.)
Procreation: I wanted to conceive a child
Social status: I wanted to enhance my social status or reputation
Revenge: I wanted my partner to feel jealous or hurt
Utilitarian reasons: I had sex for utilitarian reasons (such as burning calories, hoping to get rid of a headache or keeping warm)
Love and commitment: I wanted to feel connected to the person, express my love and commitment
Expression: I wanted to have sex in order to express my feelings such as being sorry, thankful, etc.
Self-esteem boost: I wanted to boost my self-esteem (such as feeling attractive or powerful)
Duty/pressure: I felt obligated or didn’t know how to say “no”
Mate guarding: I wanted to keep my partner from having sex with someone else.

YSEX? questionnaire (Meston and Buss, 2007) was developed using mostly heterosexual monogamous sample of college students. However, the list of reasons reported by this group may not fully satisfy the needs of non-monogamous population. Literature suggests that non-monogamous relationships provide an opportunity to meet one’s diverse needs through multiple relationships (Mitchell et al., 2014; Balzarini and Muise, 2020), which leads to a conclusion that non-monogamous individuals may have more or other needs than monogamous individuals and result in additional reasons to engage in sex compared to monogamous individuals.

Carlström and Andersson (2019) investigated the relationship between BDSM (bondage and discipline, dominance and submission, and sadism and masochism) interests and non-monogamy. They state that non-monogamy is a logical choice for people who identify as queer if they want to satisfy their kinky needs (Carlström and Andersson, 2019). Moreover, being kinky is a transgression of norms *per se*, which also facilities the transgression of the norms of monogamy (Carlström and Andersson, 2019). The study by Vilkin and Sprott (2021) supports this idea, stating that kink interests are an important motivator to engage in consensual non-monogamy. Based on these findings and having in mind people who have specific sexual interests, the following question was included in the survey questionnaire of this study:

Specific sex: “I wanted to have sex which I cannot have with my other partner (such as kink, fetish, anal, etc.)”

A study by Mogilski et al. (2017) suggest that people who have multiple partners are more likely to identify their sexuality in non-polar and non-traditional ways compared to people who have one sexual and romantic partner. Other studies explore so called “mixed orientation marriages,” which are marital unions where one of the partners is heterosexual and the other is not (i.e., gay, lesbian, bisexual) (Shao et al., 2021). Some authors suggest that bisexuality is non-transitional in nature (Diamond, 2009) and might emerge before or within the course of a marriage (Jordal, 2011). Jordal (2011) investigated marital commitment among mixed orientation couples and found out that while some couples keep their marital commitment closed (staying monogamous), other couples open their marital commitment to fulfill their bisexual needs. The latter agreement entails either a negotiated option to open the relationship (becoming non-monogamous), opening up for one of the partners, opening up for both partners (or becoming polyamorous) or including a third person for both (Jordal, 2011). Having these non-monogamous people in mind, an additional survey question has emerged:

Another gender: “I wanted to have sex with a person of an opposite gender than my other partner.”

Social psychologists propose an idea that unethical behavior can actually trigger positive affect in the cheating person (Ruedy et al., 2013). The “cheater’s high” might be explained by the notion of “forbidden fruit,” that suggest that taboo experiences or objects are more alluring and enjoyable to people than those that are not prohibited (Fishbach, 2009; Ruedy et al., 2013). This idea is represented in sexuality literature by four cornerstones of eroticism (Morin, 1996; Neves, 2021). According to Morin (1996), violation of prohibitions, like being with someone with whom one is not supposed to be, or undergoing a risk of discovery, may have high potential to experience eroticism and arousal. This idea led to another question in a survey questionnaire:

Thrill of the forbidden: “I wanted to experience the thrill of doing something forbidden.”

Together with additional questions the final version of the questionnaire consisted of 17 questions that addressed different reasons to engage in sexual activity. In addition, respondents were provided an opportunity to write their own reasons to engage in sexual activity. If respondents had more than one sexual partner, they were invited to respond to the questions of the questionnaire about two current partners. Responses were given on Likert-type scale (anchored 1 = none of my sexual experiences and 5 = all of my sexual experiences).

Both to identify if there are statistically significant differences in frequencies choosing different reasons to engage in sexual activity among monogamous and non-monogamous respondents in committed relationships and if there are gender differences in frequency of reasons to engage in sex, the authors used Mann-Whitney *U*-test.

## Results

To answer the research question if the reasons to engage in sex are different among monogamous and non-monogamous respondents in committed relationships, self-reported motivation to engage in sex due to various reasons was compared between both groups (see Table 1).

**TABLE 1:** Reasons to engage in sex among monogamous and non-monogamous respondents.

	**Monogamous respondents**			**Non-monogamous respondents (average between both partners)**				**Non-monogamous respondents (partner 1)**				**Non-monogamous respondents (partner 2)**			
	***n* = 641**			***n* = 596**				***n* = 596**				***n* = 596**			
	***M***	***SD***	***Mdn***	***M***	***SD***	***Mdn***	***U***	***M***	***SD***	***Mdn***	***U***	***M***	***SD***	***Mdn***	***U***
Stress reduction	2.41	0.9	2	2.54	0.99	2.5	180946	2.68	1.01	3	163904***	2.40	1.28	2	182999
Pleasure	3.96	0.70	4	3.90	0.73	4	185131.5	3.97	0.73	4	188926.5	3.83	1.06	4	188815
Physical desirability	3.75	0.99	4	3.78	0.83	4	190157	3.84	0.97	4	181395.5	3.72	1.11	4	189325
Experience seeking	2.41	0.89	2	3.13	0.83	3	105820.5***	2.92	0.97	3	135682,5***	3.34	1.18	4	102952***
Resources	1.10	0.4	1	1.54	0.99	1	146306***	1.54	1.05	1	153701,5***	1.53	1.07	1	156575***
Procreation	1.58	0.84	1	1.64	0.92	1.5	179408.5***	1.88	1.14	1	167752,5***	**1.41**	0.96	1	157401***
Social status	1.14	0.46	1	1.55	0.96	1	147449.5***	1.55	1.05	1	158197***	1.55	1.02	1	155527***
Revenge	1.11	0.4	1	1.47	0.94	1	157406***	1.46	0.98	1	164141***	1.48	1.01	1	162087***
Utilitarian	1.45	0.69	1	1.71	1.00	1	165634.5***	1.80	1.06	1	161048***	1.62	1.08	1	190881
Love and commitment	3.99	0.76	4	3.24	0.83	3	93549***	3.92	0.85	4	184790	**2.55**	1.42	2	84100,5***
Expression	2.38	1.04	2	2.34	0.99	2	185063.5	2.70	1.11	3	160487***	**1.97**	1.22	1	144190***
Self-esteem boost	2.31	1.04	2	2.63	1.07	2.5	158180.5***	2.57	1.13	3	167809,5***	2.69	1.28	3	158915***
Duty/pressure	1.67	0.82	1	1.81	0.92	1.5	173308**	1.91	1.02	2	169133,5***	1.71	1.04	1	183964.0
Mate guarding	1.35	0.79	1	1.60	0.99	1	162663***	1.65	1.09	1	168573***	1.55	1.04	1	179019,5*
Specific sex	1.23	0.59	1	2.21	1.02	2	77163***	1.94	1.15	1	122839,5***	2.48	1.35	2	87374,5***
Another gender	1.15	0.47	1	1.85	1.07	1	114926***	1.73	1.11	1	137172***	1.97	1.26	1	122220,5***
Thrill of the forbidden	1.61	0.77	1	2.41	1.01	2.5	101856.5***	2.12	1.11	2	143479,5***	2.71	1.32	3	100226,5***

significant differences are highlighted in bold, *p ≤ 0.05; **p ≤ 0.01; ***p ≤ 0.001.

Out of 17 reasons, the following three reasons to engage in sexual activity were most often reported by *monogamous respondents*—desire to feel connected and express love and commitment (*M* = 3.99, *SD* = 0.76; here and further answers were given on a Likert scale from 1 to 5, where “1” was “none of my experiences” and “5”—“all of my experiences”), desire to experience physical pleasure (*M* = 3.96, *SD* = 0.70) and experiencing physical desire toward a partner (*M* = 3.75, *SD* = 0.99). The next most frequent reasons to engage in sexual activity were desire to release stress anxiety, tension or to fight boredom (*M* = 2.41, *SD* = 0.9); desire to have a new sexual experience or to act out a fantasy (*M* = 2.41, *SD* = 0.89); expression of respondent’s feelings such as being sorry, thankful, etc. (*M* = 2.38, *SD* = 1.04); desire to boost self-esteem such as feeling attractive or powerful (*M* = 2.31, *SD* = 1.04). The least frequent reason among monogamous respondents to engage in sexual activity was to get resources from their partner such as promotion, money, etc. (*M* = 1.10, *SD* = 0.4).

To identify the most frequently reported reasons to engage in sexual activity among *non-monogamous respondents*, the average score of non-monogamous a respondent’s both partners was calculated for each of the reasons. Like for monogamous respondents, the most frequent reasons to engage in sexual activity for non-monogamous respondents were—desire to experience physical pleasure (*M* = 3.90, *SD* = 0.73) and physical desirability of a partner (*M* = 3.78, *SD* = 0.83). Next goes the desire to feel connected to their partner, express their love and commitment (*M* = 3.24, *SD* = 0.83) and desire to have a new sexual experience or to act out a fantasy (*M* = 3.13, *SD* = 0.83). Similarly, to monogamous, the least frequent reasons to engage in sexual activity among non-monogamous respondents were desire to get resources from that person such as promotion, money, etc. (*M* = 1.54, *SD* = 0.99) and desire to make their partner feel jealous or hurt (*M* = 1.47, *SD* = 0.94).

To identify if there are statistically significant differences in frequencies in choosing different reasons to engage in sexual activity among monogamous and non-monogamous respondents, the data was compared in three ways. First, answers of monogamous respondents and an average score of two partners of non-monogamous respondents. Second, answers of monogamous respondents and answers of non-monogamous respondents about their first partner. And finally, answers of monogamous respondents and answers of non-monogamous respondents about their second partner. The detailed differences are presented in the Table 1. Non-monogamous respondents showed significantly higher scores in most of the reasons to engage in sexual activity compared to monogamous participants, except for the cases when the reason to engage in sexual activity was respondent’s own sexual excitement or physical desirability of the partner. Non-monogamous respondents significantly more often engaged in sexual activity because they wanted a new sexual experience or to act out a fantasy (*U* = 105820.5, *p* < 0.001), because they wanted resources from their partner (*U* = 146306, *p* < 0.001), because they wanted to conceive a child (*U* = 179408.5, *p* < 0.001), because they wanted to enhance their social status (*U* = 147449.5, *p* < 0.001), also because they wanted to take revenge their other partner because of feeling jealous or hurt (*U* = 157406, *p* < 0.001), to boost their self-esteem (*U* = 158180.5, *p* < 0.001), because they wanted to keep any of their partners from having sex with someone else (*U* = 162663, *p* < 0.001), because they wanted to have sex which they cannot have with one of their partner’s such as kink, fetish, anal, etc. (*U* = 77163, *p* < 0.001), to have sex with a person of an opposite gender (*U* = 114926, *p* < 0.001), and to experience the thrill of doing something forbidden (*U* = 101856.5, *p* < 0.001). Non-monogamous respondents also indicated to have sex more often for utilitarian reasons such as burning calories, hoping to get rid of a headache or keeping warm (*U* = 165634.5, *p* < 0.001) compared to monogamous participants. However, this is at the expense of having sex for utilitarian reasons more often with their primary partner, not secondary. Non-monogamous respondents less often have sex to express their love and commitment (*U* = 93549, *p* < 0.001) compared to monogamous respondents, this is mostly at the expense of the secondary partner with whom they have sex significantly less often for this reason. Although there is no difference in the frequency to engage in sexual activity to express feelings such as being sorry, thankful, etc. when comparing all partners of non-monogamous respondents to monogamous respondents, the differences are pronounced on the partner’s level. Non-monogamous significantly more often engage in sexual activity with their primary partner to express their feelings (*U* = 160487, *p* < 0.001) and less often with their secondary partner (*U* = 144190, *p* < 0.001). Non-monogamous respondents also showed higher frequency to engage in sexual activity out of obligation with their primary partner (*U* = 169133.5, *p* < 0.001), but not the secondary partner. And although there are no significant differences to engage in sexual activity to release stress, anxiety, tension or to fight boredom among monogamous and non-monogamous respondents, on the partners’ level non-monogamous respondents significantly more often engaged in sexual activity to release stress with their primary partners (*U* = 163904, *p* < 0.001).

To sum up, the desire for physical pleasure and physical desirability of the partner are two universal and most frequent reasons to engage in sex both for monogamous and non-monogamous respondents. The next most reported reason to engage in sex for both groups is desire to express love and commitment. However, this is a more pronounced reason in relation to non-monogamous respondent’s primary, but not secondary partner. Both monogamous and non-monogamous groups reported revenge, desire to get resources and desire to enhance social status as the last frequent reasons to engage in sex. Though, non-monogamous respondents reported higher frequency to engage in sex almost for all provided reasons. Moreover, non-monogamous respondents significantly more often engaged in sex seeking new experience, willing to boost their self-esteem, wanting specific form of sex and wanting to experience the thrill of the forbidden.

The authors also investigated gender differences in reasons to engage in sex. When comparing all male participants to all female participants of the study irrespectively of their non/monogamy status, significant differences were found in 9 out of 17 reasons to engage in sex (see Table 2). Men engaged in sex more often than women for eight reasons (in the order from the most to less frequent)—the physical desirability of the partner (*U* = 358847.5, *p* < 0.001), seeking new sexual experience (*U* = 336164,0, *p* < 0.001), to reduce stress (*U* = 345575, *p* < 0.001), wanting to experience the thrill of the forbidden (*U* = 313963.5, *p* < 0.001), looking for specific type of sex like kink or fetish (*U* = 328740.5, *p* < 0.001), desire to enhance their social status (*U* = 347172.5, *p* < 0.001), to get resources from a person (*U* = 377345.5, *p* = 0.001), to make a partner feel jealous or hurt (revenge) (*U* = 375197.5, *p* < 0.001). Women had sex more often than men for one reason—to feel connected to the person, express love and commitment (*U* = 351142.5, *p* < 0.001).

**TABLE 2:** Reasons to engage in sex among men and women.

**Reasons to engage in sex**	**Males**			**Females**			
	***n* = 630**			***n = 587***			
	***M***	***SD***	***Mdn***	***M***	***SD***	***Mdn***	***U***
Stress reduction	2.6	1.1	3.0	2.3	1.0	2.0	345575***
Pleasure	3.9	0.8	4.0	3.9	0.8	4.0	391617.5
Physical desirability	3.9	0.9	4.0	3.7	1.1	4.0	358847.5***
Experience seeking	3.0	1.1	3.0	2.7	1.1	3.0	336164***
Resources	1.5	1.0	1.0	1.3	0.8	1.0	377345.5***
Procreation	1.7	1.1	1.0	1.6	0.9	1.0	380960**
Social status	1.6	1.0	1.0	1.2	0.7	1.0	347172.5***
Revenge	1.4	1.0	1.0	1.3	0.7	1.0	375197.5***
Utilitarian	1.7	1.0	1.0	1.5	0.9	1.0	389879.5
Love and commitment	3.4	1.3	4.0	3.6	1.2	4.0	351142.5***
Expression	2.4	1.2	2.0	2.3	1.1	2.0	388168.0
Self-esteem boost	2.5	1.2	2.0	2.5	1.1	2.0	395223.0
Duty/pressure	1.7	1.0	1.0	1.8	0.9	2.0	385564.5
Mate guarding	1.6	1.0	1.0	1.5	0.9	1.0	387413.5
Specific sex	2.1	1.3	2.0	1.6	1.1	1.0	328740.5***
Another gender	1.7	1.1	1.0	1.6	1.0	1.0	397285.5
Thrill of the forbidden	2.4	1.2	2.0	1.9	1.1	2.0	313963.5***

significant differences are highlighted in bold, *p ≤ 0.05; **p ≤ 0.01; ***p ≤ 0.001.

To understand gender differences further in respect of reasons to engage in sex, the authors compared gender differences considering their non/monogamy status. Comparisons were made within the following groups: monogamous men vs. monogamous women, non-monogamous men vs. non-monogamous women, monogamous men vs. non-monogamous men, monogamous women vs. non-monogamous women. Similarly, to previous findings, there are no differences in reasons to engage in sex if a person was seeking physical pleasure. While men engage in sex more often to reduce stress (*U* = 345575, *p* < 0.001), this is not mitigated by monogamy status as monogamous women showed no differences compared to non-monogamous women and monogamous men showed no differences compared to non-monogamous men in this respect. Similar findings apply to the desire to engage in sex for the reason of physical desirability of a partner, differences are pronounced on the gender level (men more often engage in sex for this reason than women, *U* = 358847.5, *p* < 0.001), but are not dependent on monogamy status. Interestingly, while the results showed no differences between men and women in engaging in sex to keep their partner from having sex with someone else, there are differences in mate guarding when monogamy status is applied—monogamous men engage in sex less often than women to guard their mates (*U* = 43946.0, *p* = 0,004), at the same time non-monogamous men engage in sex more often than women to guard their mates (*U* = 149918.5, *p* = 0.001) and, logically, non-monogamous men engage in sex more often than monogamous men to guard their mate (*U* = 79836.5, *p* < 0.001). This study did not find any differences among monogamous and non- monogamous women in this respect—monogamy status does not lead women to have less or more sex to keep their partners from straying. Both monogamous and non-monogamous men more often than women engage in sex to experience a specific type of sex, like kink or fetish (monogamous: *U* = 44831.5, *p* = 0,008; non-monogamous: *U* = 140096, *p* < 0.001) and non-monogamous respondents of both genders engage more often in this type of sex than their monogamous counterparts. See Table 3 for more details on the monogamy status and gender relationship with reasons to engage in sex.

**TABLE 3:** Reasons to engage in sex among monogamous and non-monogamous men and women.

																													
	**Non-monogamous respondents**							**Monogamous respondents**							**Males**							**Females**							
	**Males**			**Females**				**Males**			**Females**				**Monogamous**			**Non-monogamous**				**Monogamous**			**Non-monogamous**				
	***M***	***SD***	***Mdn***	***M***	***SD***	***Mdn***	***U***	***M***	***SD***	***Mdn***	***M***	***SD***	***Mdn***	***U***	***M***	***SD***	***Mdn***	***M***	***SD***	***Mdn***	***U***	***M***	***SD***	***Mdn***	***M***	***SD***	***Mdn***	***Mdn***	***U***
																													
Stress reduction	2.7	1.2	3.0	2.4	1.1	2.0	140548**	2.5	0.9	3.0	2.3	0.9	2.0	42543.5**	2.5	0.9	3.0	2.7	1.2	3.0	91341.5	2.3	0.9	2.0	2.4	1.1	2.0	2.0	82128.0
Pleasure	3.9	0.9	4.0	3.9	0.9	4.0	160613.0	4.0	0.7	4.0	3.9	0.7	4.0	46820.0	4.0	0.7	4.0	3.9	0.9	4.0	96664.0	3.9	0.7	4.0	3.9	0.9	4.0	4.0	81714.5
Physical desirability	3.9	1.0	4.0	3.7	1.1	4.0	151002.5**	3.9	0.8	4.0	3.6	1.1	4.0	41095***	3.9	0.8	4.0	3.9	1.0	4.0	95728.0	3.6	1.1	4.0	3.7	1.1	4.0	4.0	78504.0
Experience seeking	3.2	1.1	3.0	3.0	1.1	3.0	145735.5***	2.5	0.9	2.0	2.3	0.9	2.0	43559**	2.5	0.9	2.0	3.2	1.1	3.0	59125***	2.3	0.9	2.0	3.0	1.1	3.0	3.0	54039***
Resources	1.6	1.1	1.0	1.4	1.0	1.0	151955.5**	1.1	0.3	1.0	1.1	0.4	1.0	46499.5**	1.1	0.3	1.0	1.6	1.1	1.0	73717***	1.1	0.4	1.0	1.4	1.0	1.0	1.0	71502***
Procreation	1.7	1.1	1.0	1.5	1.0	1.0	149858**	1.6	0.8	1.0	1.6	0.9	1.0	48452.0	1.6	0.8	1.0	1.7	1.1	1.0	96235.0	1.6	0.9	1.0	1.5	1.0	1.0	1.0	75267.0
Social status	1.7	1.1	1.0	1.3	0.8	1.0	138167.5***	1.2	0.5	1.0	1.1	0.4	1.0	47287.0	1.2	0.5	1.0	1.7	1.1	1.0	74608,5***	1.1	0.4	1.0	1.3	0.8	1.0	1.0	74325***
Revenge	1.6	1.1	1.0	1.4	0.9	1.0	151184.5**	1.1	0.4	1.0	1.1	0.4	1.0	47774.0	1.1	0.4	1.0	1.6	1.1	1.0	78799***	1.1	0.4	1.0	1.4	0.9	1.0	1.0	74476.0
Utilitarian	1.8	1.1	1.0	1.6	1.0	1.0	151650.5**	1.4	0.7	1.0	1.5	0.7	1.0	43842.5**	1.4	0.7	1.0	1.8	1.1	1.0	81366,5***	1.5	0.7	1.0	1.6	1.0	1.0	1.0	81798.0
Love and commitment	3.2	1.3	4.0	3.3	1.4	4.0	152348**	3.9	0.8	4.0	4.1	0.7	4.0	43576**	3.9	0.8	4.0	3.2	1.3	4.0	68422***	4.1	0.7	4.0	3.3	1.4	4.0	4.0	59722,5***
Expression	2.4	1.3	2.0	2.2	1.1	2.0	152803.5**	2.4	1.1	2.0	2.4	1.0	2.0	48106.0	2.4	1.1	2.0	2.4	1.3	2.0	96826.0	2.4	1.0	2.0	2.2	1.1	2.0	2.0	74906**
Self-esteem boost	2.6	1.2	3.0	2.7	1.2	3.0	162568.5	2.2	1.1	2.0	2.4	1.0	2.0	43407.5	2.2	1.1	2.0	2.6	1.2	3.0	78225***	2.4	1.0	2.0	2.7	1.2	3.0	3.0	72209**
Duty/pressure	1.9	1.1	1.0	1.8	1.0	1.0	157757.0	1.5	0.7	1.0	1.8	0.9	2.0	37239***	1.5	0.7	1.0	1.9	1.1	1.0	79625***	1.8	0.9	2.0	1.8	1.0	1.0	1.0	75930,5**
Mate guarding	1.7	1.1	1.0	1.5	0.9	1.0	149918.5**	1.2	0.6	1.0	1.4	0.9	1.0	43946**	1.2	0.6	1.0	1.7	1.1	1.0	79836,5***	1.4	0.9	1.0	1.5	0.9	1.0	1.0	82206.5
Specific sex	2.4	1.3	2.0	2.0	1.2	2.0	140096***	1.3	0.7	1.0	1.2	0.5	1.0	44831.5**	1.3	0.7	1.0	2.4	1.3	2.0	51266,5***	1.2	0.5	1.0	2.0	1.2	2.0	2.0	48863***
Another gender	1.9	1.2	1.0	1.9	1.1	1.0	158753.0	1.1	0.5	1.0	1.2	0.5	1.0	46819.0	1.1	0.5	1.0	1.9	1.2	1.0	66240***	1.2	0.5	1.0	1.9	1.1	1.0	1.0	53494,5***
Thrill of the forbidden	2.6	1.2	3.0	2.2	1.2	2.0	130841.5***	1.7	0.8	1.0	1.5	0.7	1.0	43561.5	1.7	0.8	1.0	2.6	1.2	3.0	56359***	1.5	0.7	1.0	2.2	1.2	2.0	2.0	59232***

significant differences are highlighted in bold, *p ≤ 0.05; **p ≤ 0.01; ***p ≤ 0.001.

To sum up, physical pleasure seems to be a universal reason to engage in sex and does not depend either on monogamy status or gender. Men more often engage in sex to reduce stress and because of physical desirability of a partner, but it is not mitigated by monogamy status. Non-monogamous men tend to engage in sex more often to keep their partner from having sex with someone else compared both to women and monogamous men. There are no differences in this respect among monogamous and non-monogamous women.

## Discussion and Implications

The purpose of this study was to investigate motivation to engage in sex among monogamous and non-monogamous adults. As a result, this research has provided various inputs for better understanding of human sexuality.

First, it expanded the number of reasons why people engage in sex. Previously, other studies identified a limited number of reasons to engage in sex and were limited to obvious motives—emotional closeness, physical pleasure, and reproduction. Meston and Buss (2007) made a significant breakthrough in understanding motivations to engage in sex by making a comprehensive list of 142 reasons grouped into 13 factors. This study complemented previous body of research by three additional reasons—desire for specific sex, desire for sex with a partner of another gender, and desire to experience the thrill of the forbidden. These reasons are especially important considering non-monogamous population.

Second, this research has enhanced the current body of knowledge about motivations to engage in sex among monogamous and non-monogamous populations. Previous body of research has shown that the most frequent reasons to engage in sex are related to pleasure and physical desirability (Meston and Buss, 2007; Wood et al., 2014). This research has demonstrated that while two most frequent reasons to engage in sex (pleasure and physical desirability) are universal both for monogamous and non-monogamous respondents, there are significant differences in all other reasons to engage in sex among monogamous and non-monogamous respondents. On top of that, non-monogamous respondents report significantly higher frequency of engagement in sex for most reasons. However, further research is necessary to identify whether monogamous and non-monogamous people are fundamentally different in their sexual needs or whether it is their relationship arrangement that provides the environment for different sexual needs and desires.

This research has also addressed gender differences in reasons to have sex. Differences in reasons to engage in sex between men and women were investigated in the original YSEX? study by Meston and Buss (2007). However, the sample in the research of Meston and Buss (2007) was represented mainly by psychology students (96% between the ages of 18 and 22) and may not be fully applicable to general population. This study clearly confirms findings in differences in half of the reasons to engage in sex reported by Meston and Buss (2007)—stress reduction, physical desirability, experience seeking, resources, social status, revenge. However, this study has found that women engage in sex more often to express love and commitment, while Meston and Buss (2007) found no differences among men and women in this respect. This study also found no differences in reasons to engage in sex between men and women for the reason of physical pleasure, though Meston and Buss (2007) reported that men engage in sex more often than women for this reason. This may be explained by the older age of participants of current study (*M* = 27,78 years, *SD* = 7.53, range = 18–62), when women might feel more empowered to pursue physical pleasure without feelings of love and commitment.

Last, but not least, by illuminating the variety of reasons why couples engage in non-monogamous sexual activity, this research is also beneficial for practicing psychotherapists. Brandon (2016) argues that it is challenging to help their clients until the psychotherapist reaches some level of understanding and personal acceptance that people arrange their intimate relationships in various ways. Thus, Brandon (2016) invites psychotherapists not to ignore the challenges of monogamy, but to investigate them deeper and further. Schechinger et al. (2018) emphasize the need for more research on non-monogamy and training for practitioners who work with consensually non-monogamous clients. Findings of this and other research works on non-monogamy may be helpful in aiding professionals to help their clients to responsibly manage their non-monogamous relationships.

## Limitations and Future Directions

One of the strengths of this study is that it gathered responses of a large and international sample of non-monogamous respondents who are notoriously difficult to reach. The non-monogamous sample is represented by people who practice different monogamy agreements, including non-consensually non-monogamous adults, swingers, polyamorous adults, and adults in open relationships. However, study has its limitations. While some non-monogamous respondents may have several concurrent sexual partners, this study focused only on two partners if the respondent indicated being non-monogamous. Only half of multi-partner participants of the study by Mogilski et al. (2020) reported having two partners, while one quarter had three concurrent partners and one fifth—four or more partners). Studying reasons to engage in sexual activity with each of respondent’s sexual partners (if more than two) may give us a better understanding of reasons to engage in sexual activity with the whole cohort of partners. However, in the context of this study asking respondents about their all sexual partners would be complicated and time consuming for the respondents themselves. Further studies should look into potential differences in reasons to engage in sex with respondents’ different partners if there is more than one partner.

This study asked participants to respond about their current relationship status and did not investigate whether a respondent engaged in other either monogamous or non-monogamous relationships in the past.

Even though the research did not uncover additional reasons to engage in sexual activities by collecting respondents’ own write-in suggestions, there still may be other reasons to engage in sex than the ones used for this study that are relevant to non-monogamous population.

Psychological phenomena do not exist in a vacuum, and this applies to monogamy and non-monogamy. Thus, additional research is needed to understand how personality traits, economic, social and relationship factors impact people’s motivation to engage in monogamous or non-monogamous sex. Some authors suggest that religiosity may be correlated with sexual behavior (Ahrold et al., 2011; McFarland et al., 2011). Thus, in further research the data should be controlled for religious beliefs, spirituality and cultural attitudes.

## Data Availability Statement

The raw data supporting the conclusions of this article will be made available by the authors, without undue reservation.

## Ethics Statement

The studies involving human participants were reviewed and approved by the Ethics Committee for Humanities and Social Sciences research involving human participants, University of Latvia. The patients/participants provided their written informed consent to participate in this study.

## Author Contributions

AK contributed to conception and design of the study, organized the database, performed the statistical analysis, and wrote the first draft and sections of the manuscript. Both authors contributed to manuscript revision, read, and approved the submitted version.

## Conflict of Interest

The authors declare that the research was conducted in the absence of any commercial or financial relationships that could be construed as a potential conflict of interest.

## Publisher’s Note

All claims expressed in this article are solely those of the authors and do not necessarily represent those of their affiliated organizations, or those of the publisher, the editors and the reviewers. Any product that may be evaluated in this article, or claim that may be made by its manufacturer, is not guaranteed or endorsed by the publisher.
